# Testing multiple hypotheses through IMP weighted FDR based on a genetic functional network with application to a new zebrafish transcriptome study

**DOI:** 10.1186/s13040-015-0050-8

**Published:** 2015-06-17

**Authors:** Jiang Gui, Casey S. Greene, Con Sullivan, Walter Taylor, Jason H. Moore, Carol Kim

**Affiliations:** 1 Department of Biomedical Data Science, Geisel school of medicine, Dartmouth College, Hanover, NH USA; 2 Department of Genetics, Geisel school of medicine, Dartmouth College, Hanover, NH USA; 3 Department of Molecular and Biomedical Sciences, University of Maine, Orono, ME USA; 4 Dartmouth-Hitchcock Medical Center, 883 Rubin Bldg, HB7927, One Medical Center Dr., Lebanon, NH USA; 5 Graduate School of Biomedical Science and Engineeering, University of Maine, Orono, ME USA; 6 Department of Biostatistics and Epidemiology, The Perelman School of Medicine, University of Pennsylvania, Philadelphia, PA USA

**Keywords:** False discovery rate, Family-wise error rate, Genomic studies, Data integration

## Abstract

In genome-wide studies, hundreds of thousands of hypothesis tests are performed simultaneously. Bonferroni correction and False Discovery Rate (FDR) can effectively control type I error but often yield a high false negative rate. We aim to develop a more powerful method to detect differentially expressed genes. We present a Weighted False Discovery Rate (WFDR) method that incorporate biological knowledge from genetic networks. We first identify weights using Integrative Multi-species Prediction (IMP) and then apply the weights in WFDR to identify differentially expressed genes through an IMP-WFDR algorithm. We performed a gene expression experiment to identify zebrafish genes that change expression in the presence of arsenic during a systemic *Pseudomonas aeruginosa* infection. Zebrafish were exposed to arsenic at 10 parts per billion and/or infected with *P. aeruginosa*. Appropriate controls were included. We then applied IMP-WFDR during the analysis of differentially expressed genes. We compared the mRNA expression for each group and found over 200 differentially expressed genes and several enriched pathways including defense response pathways, arsenic response pathways, and the Notch signaling pathway.

## Introduction

With the rapid development of novel high-throughput deep sequencing technology, the study of functional transcriptomes has changed dramatically. Compared to microarray-based measurements, RNA-sequencing (RNA-seq) technology can profile RNA transcript abundance within greater depth and accuracy. It can effectively detect alternative splicing variants and novel transcripts and does not require an assembled genome sequence. Study [1] compared high-throughput sequencing data in Illumina and Affymetrix platform and showed that sequencing data are highly replicable, with much smaller technical variation when compared to microarray data. In many cases, it is sufficient to sequence each mRNA sample only once.

RNA-seq analysis raises significant challenges when tens of thousands of transcripts are tested at the same time in order to find those that are differentially expressed under given conditions. Multiple testing correction is required to adequately control type I error, and frequently Bonferroni correction [2] is used to adjust for multiple testing. The Bonferroni correction is highly stringent, and this correction controls the error rate by adjusting the significance threshold to maintain the specified p-value threshold across all tests. This strict correction is less appropriate for genomic data analysis, where the cost of a false positive is relatively low, the number of observations is relatively low, and the number of tests is high. Alternative criteria that offer a lower false negative rate (*i.e.* missed findings) are more appropriate in this setting. The false discovery rate (FDR) was proposed by Benjamini and Hochberg [3] for such settings. This test is more lenient than attempts to control the family wise error rate (FWER), and it allows adequate control of false positives during genomic data analysis and has become widely used [4–7].

While FDR provides an improvement for Type II when compared to FWER-based methods, the FDR method still suffers from reduced power when there are a large number of tests. The incorporation of weights representing the strength of existing evidence was developed by Genovese *et al.* [8] and provides a means to further improve power. Genovese *et al.* proved that weight assignments consistent with the rule that the sum of the weights equaling the total number of tests resulted in effective control of the FDR at the nominal level (*i.e.* with WFDR at a nominal level of 0.05, 5 % of the discoveries would be false positives just as with the unweighted FDR). In the field of biology, researchers have access to substantial information about biology from both published experiments and existing biological data. Leveraging this knowledge to improve the power of genomic tests would provide significant advantages; and FDR weighting methods that combine biological information and knowledge with gene expression measurements to more effectively identify differentially expressed genes represent an area of unmet need.

The Integrative Multi-species Prediction [9, 10] webserver has collected over 2,000 microarray datasets from the NCBI Gene Expression Omnibus (GEO), large collection of biochemical experiments from the Database of Protein and Genetic Interactions (BioGRID), tissue-specificity and phenotype characterizations from The Zebrafish Model Organism Database (ZFIN) and other sources for seven organisms. IMP integrates these data into functional relationship networks using naïve Bayesian classifiers. Functional networks represent an efficient and comprehensive summary of existing publicly available data. Specifically, in each functional network, each node represents a gene and each edge between two genes is the posterior probability that the two genes are involved in the same process or pathway [11]. IMP is available through a web server (http://imp.princeton.edu/). IMP provides gene-gene functional relationship networks from multiple organisms including zebrafish. The IMP networks have been used to identify novel genes involved in the development of left-right asymmetry in zebrafish embryos, which were validated through knockdown experiments [10]. While these networks have demonstrated utility for guiding targeted experiments, methods that allow researchers to use these networks to effectively analyze new large-scale experiments would address a currently unmet need.

In this paper, we develop and evaluate a novel IMP-WFDR method that uses state-of-the-art genetic network information to infer appropriate weights for WFDR and adjusts the p-values from simultaneous tests to correct for multiple testing issue.

The genetic disorder cystic fibrosis (CF) results from a loss of function variant in the *cystic fibrosis transmembrane conductance regulator* (*CFTR*) gene. CF individuals have difficulty with airway clearance, and most will develop chronic *P. aeruginosa* [12] infections. While the underlying cause of CF is genetic, the environment is expected to play a role in the many factors of the disease. For example, arsenic has recently been shown to alter CFTR function [13, 14]. We developed a zebrafish model for CF and found that both *cftr* and arsenic individually affect innate immunity [15]. Here we identify gene expression changes associated with the innate immune response in the context of arsenic exposure. We performed RNA sequencing of control zebrafish as well as those exposed to arsenic at levels seen in the environment (2 ppb and 10 ppb) and those experiencing a chronic *P. aeruginosa* infection. We apply our newly developed IMP-WFDR method to detect genes that were differentially expressed upon exposure to arsenic and/or infection with *P. aeruginosa*. The IMP-WFDR method integrates large data compendia through functional networks and focuses these data on the analysis of a new genome-scale experiment to better identify relevant candidates.

## Methods

### IMP-WFDR method

Genovese *et al.* [8] proposed a simple weighted FDR procedure in which non-negative weights W_i_ are assigned to each p-value such that ∑_i = 1_^m^W_i_ = m. Here m refer th the number of total genes. The BH procedure is applied directly to Q_i_ = P_i_/W_i_, where P_i_ denotes the unweighted p-value. They have proven that weighted FDR controls FDR at the nominal level.

**Fig. 1:**
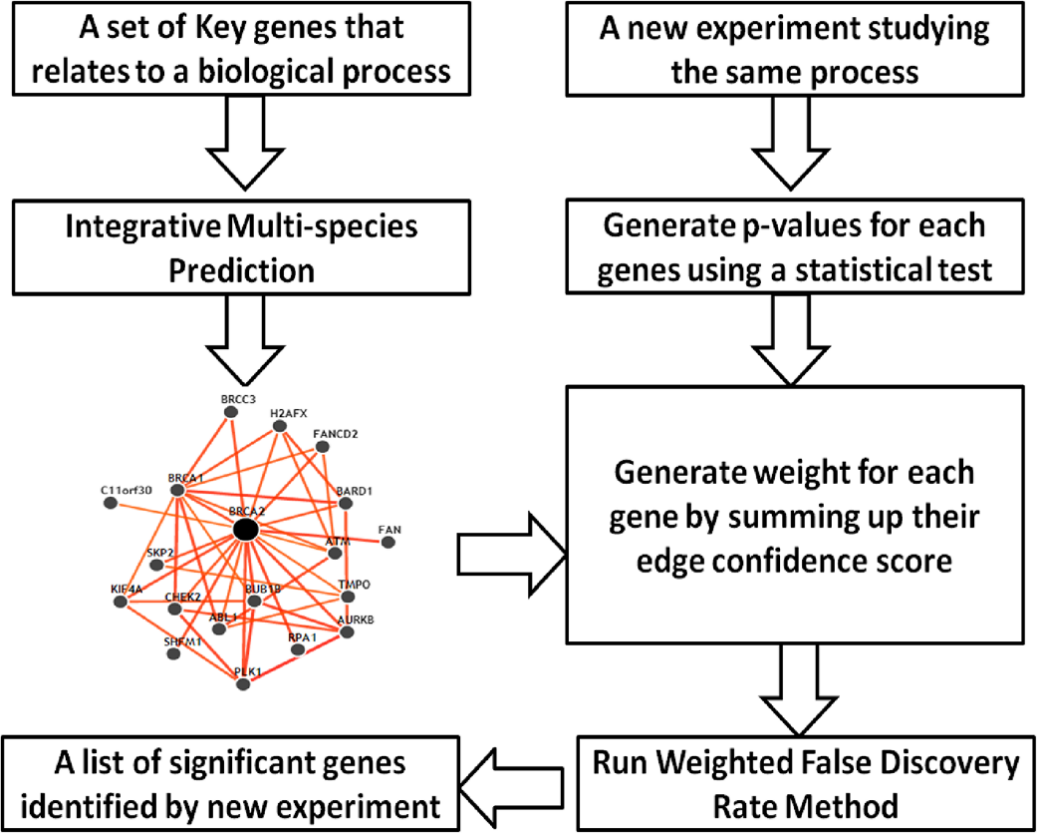
Demonstration of WFDR-IMP procedure

Given the freedom of assigning weights to p-values, weighted FDR provides an excellent platform to incorporate expert biological knowledge of each gene’s biological function. Because we already have substantial knowledge about biology, we can use this procedure to most strongly focus on genes that represent likely candidates. If we assign weights only to the strongest candidates (*i.e.* those with known literature involvement), we limit our ability to make novel discoveries. If we assign weights evenly to all genes, we do not provide adequate weight to genes with some support. Consequently functional networks, which integrate publicly available data using biological knowledge, provide both the opportunity to make new discoveries while also allowing us to focus on candidates that are more promising than randomly selected genes [16, 17]. We use functional networks from IMP to assign weights and apply weighted FDR to adjust for multiple testing. The algorithm is depicted in Fig. 1 with the following steps:

- Identify a small set of key genes that relate to a biological process of interest.
- Input the set of gene names into IMP (https:// imp.princeton.edu) and identify networks that linked to those key genes.
- For each gene *i* in the network, calculate the total relationship confidence _i_, TRC_i_, which is the likelihood of connecting with other genes in the network. It can be calculated by summing up the confident scores of the edges linking to gene *i.*
- Set W_i_ ∝ TRC_i_ and apply weighted FDR to adjust for multiple testing.

### Zebrafish experiment and RNA-seq dataset

**Fig. 2:**
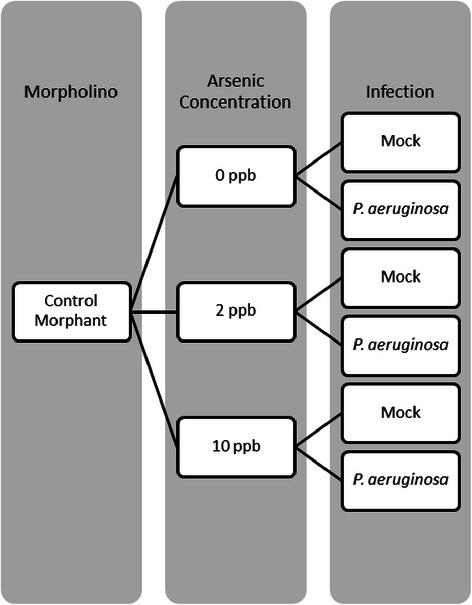
Zebrafish experiment

There is a growing interest in establishing CF-environment linkages. Using our zebrafish model, we have recently shown that the *cftr* gene responsible for CF and arsenic each separately mediate aspects of innate immunity [15, 18–20]. We hypothesize that arsenic disrupts the innate immune response. In our previous studies, we were establishing linkages that show how arsenic affects the innate immune response through its effects on Cftr function. Patients with CF typically succumb to infection by the Gram-negative bacterium *P. aeruginosa*. We recently established a zebrafish model for *P. aeruginosa* infection and have applied it to our Cftr morphant fish [20]. We have shown that Cftr morphant fish are indeed more susceptible to *P. aeruginosa* infection. In this study, we generated eighteen groups of zebrafish, each with three replicates, based upon [1] exposure to different levels of arsenic, and [2] infection with *P. aeruginosa* (Fig. 2) for each of the three time points tested (6, 12, and 18 h post-P. aeruginosa infection). At 6, 12, and 18 h post-infection, larvae were collected and homogenized in Trizol reagent (Life Technologies, Carlsbad, CA)., using a motorized mortar and pestle. Total RNA was extracted, as per manufacturer’s instructions RNA integrity was ensured using Nano 6000 RNA chips with the Agilent 2100 Bioanalyzer (Santa Clara, CA). RNA concentrations were determined with the Nanodrop UV/Vis spectrophotometer (Wilmingtion, DE). We constructed libraries with the Illumina TruSeq v2 high throughput library construction procedure (Illumina Inc.) using one microgram of total RNA (RIN>7) and validated library construction using the Agilent Bioanalyzer 2100 followed by quantitation using the Qubit HS DNA assay and qPCR kit for Illumina (Kapa Biosystems Inc). Libraries were diluted using the Qubit or qPCR information and sequenced on the HiSeq 2000 (Agilent). The RPKM (reads per kilobase per million) value is calculated for each gene using CLC genome bench 4.5.

### Weight assignment for zebrafish experiment

To calculate weights for each gene using the IMP-WFDR procedure, we first identified 14 important genes that are associated with arsenic exposure (*fn1*, *notch1a*, *notch1b*, *pik3r1*, *akt2*, *ass1*, *nfkb2*, *foxo5*) and immune response (*il1b*, *tnfa*, *il8*, *mmp9*, *irak3*, *ifnphi1*), based on prior biological knowledge.. We queried IMP for these genes and obtained the subnetwork of all genes connected to these 14 starting genes. Out of a total of 27,834 genes, IMP was able to identify 18,803 genes in the subnetwork. The distribution of the genes’ connectivity score is right skewed and 75 % of genes’ TRC are under 0.01 with only 32 genes have a TRC greater than 1. The TRC for the 14 query genes is ranging from 0.16 to 419 with a mean of 72 and standard deviation of 113. Gene ‘*fn1*’ received a TRC of 419 which is also the largest of all genes. This is not surprising because the query genes are served as hubs for the IMP network. To stabilize the variance, we tried “log + 1” transformation for all TRCs and divided the mean value to generate the weight for Zebrafish experiment.

## Results

We developed the novel IMP-weighted FDR (IMP-WFDR) procedure to assign FDR weights. We applied this method to an RNA-seq experiment that tests an organism’s response to exposure to pathogens and arsenic, and found that IMP-WFDR effectively identified important players.

### RNA-seq data analysis results

We calculated p-values for each treatment by comparing mean RPKM (reads per kilobase per million) between PBS control samples and *P. aeruginosa*-treated samples while varying the arsenic exposure at 0 and 10 ppb concentrations at 6 or 12 h after treatment. We applied the IMP-WFDR procedure to generate weights. We compared FDR using Benjamin and Hochberg’s (BH) procedure and IMP-WFDR to adjust all p-values and used 0.2 as a cut-off. There were 4 of the 8 comparisons where at least one gene’s adjusted p-value was under 0.2. To avoid the selection bias on query genes, we set the weight for all query genes to 1 and performed a sensitivity analysis. Table 1 shows the number of findings identified by IMP-WFDR, sensitivity analysis and FDR. IMP-WFDR outperforms FDR in 3 comparisons. This indicated that IMP-WFDR can greatly improve power in detecting differentially expressed genes. The result of the sensitivity analysis is slightly better than IMP-WFDR result because a few extreme large weights are removed and distributed to the remaining genes so that more genes are upweighted. Indeed, IMP-WFDR analysis allowed us to identify numerous genes in each of the comparison groups that had previously been associated with arsenic exposure and/or immune challenge. Most of these same genes were not identifed by FDR.

**Table 1:** Compare IMP-WFDR and FDR method in zebrafish experiment

Arsenic 0 vs 10 ppb for PBS samples at 6H		Arsenic 0 vs 10 ppb for *P. aeruginosa* at 6H		PBS vs *P. aeruginosa* for Arsenic=0 ppb at 12H		PBS vs *P. aeruginosa* for Arsenic=10 ppb at 6H	
IMP-WFDR	FDR	IMP-WFDR	FDR	IMP-WFDR	FDR	IMP-WFDR	FDR
116 (143)	25	121 (129)	10	157 (167)	1	12 (9)	35

The numbers in bracket is the result from sensitivity analysis that set the weight for all query genes to 1

**Fig. 3:**
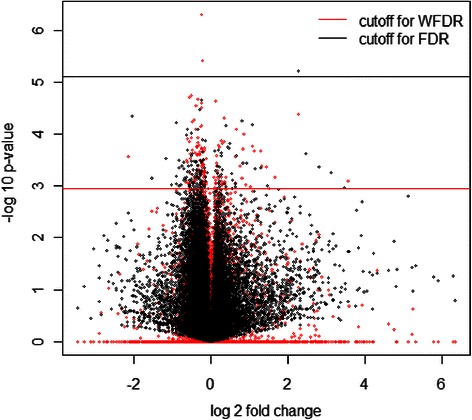
Volcano plot that overlay the p-values (black) and weighted p-values (red) from comparison of arsenic 0 vs 10 ppb for PBS samples at 6 h after treatment in zebrafish experiment

To further understand the effect of weight, we generate a volcano plot (Fig. 3) that overlay the p-values and weighted p-values from comparison of arsenic 0 vs 10 ppb for PBS samples at 6 h after treatment in zebrafish experiment. We select this comparison because it has the largest difference in number of findings. IMP-WFDR change the distribution of p-values dramatically by down-weight most of the p-values to 1 and upweight the rest. The smallest p-values for both case is at 10^(−5) level, but weighted p-value have over 140 at 10^(−3) level that will be enough to meet FDR 0.2 threshold. On other hand original p-values only have one pass FDR 0.2 threshold.

From the genes identified by IMP-WFDR, 36 genes were identified in more than one comparison. We anticipate that these genes are likely to play an important role in regulating responses to pathogens such as *P. aeruginosa* in the context of environmental exposures such as arsenic. We calculated functional enrichment p-values using this set of genes [9] to identify biological processes that were enriched within this group. Table 2 shows these processes. It is interesting to note that we identified 2 out of 6 genes (*ifnphi1, mmp9*) that are known to regulate the immune response in zebrafish. The *ifnphi1* gene encodes a type I interferon most often associated with the antiviral ressponse [21]. The *mmp9* gene encodes a collagenase that degrades extracellular matrix proteins and facilitates the migration of immune cells like neutrophils [22].

**Table 2:** GSEA results for overlapping genes

Biological process	Network Freq.	Genome Freq.	Adjusted p-values	Genes
Notch signaling pathway	19.4 % (7/36)	0.8 % (49/6131)	8.38E-06	*notch1b, jag2 gro2, dlb, dla, dld, jag1a*
regulation of defense response	5.6 % (2/36)	0.0 % (3/6131)	2.92E-02	*ifnphi1, mmp9*
regulation of immune effector process	5.6 % (2/36)	0.1 % (4/6131)	3.87E-02	*ifnphi1, mmp9*
response to arsenic containing substance	5.6 % (2/36)	0.1 % (5/6131)	4.82E-02	*il1b, ifnphi1*

The differential expression of the *ifnphi1* gene recapitulates findings that that mRNA expression of the antiviral cytokine interferon (*ifn*) was differentially expressed in arsenic-exposed zebrafish before and after viral infection in a previous study [14]. It is also important to note that the unweighted FDR procedure fails to identify these differentially expressed genes in any of the 4 comparisons. However, IMP-WFDR identifies the pair in 3 out of the 4 comparisons. This indicates that our proposed method can identify more biologically relevant genes than an unweighted FDR method, which ignores existing biological data and knowledge.

## Conclusion and discussion

We have developed a novel IMP-WFDR method that derives weights from a state-of-the-art data integration algorithm and incorporates them in WFDR to more effectively account for multiple test given the context of available biological data and knowledge. IMP has previously been used to guide morpholino assays in zebrafish [9] and our IMP-WFDR procedure greatly extends IMP’s application by improving the analysis of genomic experiments through such functional networks. We demonstrate through both a simulation study and analysis of RNA-seq data that our proposed method identifies more differentially expressed genes than unweighted FDR or Bonferroni correction, while maintaining appropriate control of the false discovery rate. We used RPKM to obtain p-values as our primary goal was to study IMP Weighted FDR, but in practice edgeR [23] or DEseq [6] can be used to calculate p-values for RNA-Seq reads.

The advantage of IMP-WFDR is that it can effectively incorporate expert biological knowledge from published experiments and existing biological data as weights and control false discovery rate to adjust for multiple testing. Roeder and Wasserman proposed a two-valued weighting scheme [24] to up weight all p-values in a pre-specified priority list and down-weight the rest. The drawback of this approach is that it demotes novel findings since all the unknown genes are down-weighted and have less of a chance to be detected than in the un-weighted case. Rather than up- weight only genes in the priority list, IMP-WFDR obtains its weight based on the functional subgenetic network defined by the priority genes. Thus, the choice of weight is much less dependent on the given priority list. From the Table 1, we see that there were over 200 genes identified by the IMP-WFDR approach while the input list contains only 14 genes. The sensitivity analysis results also support this conclusion. Despite the advantages of IMP-WFDR, it is important to recognize disadvantages of this and other weighted FDR approaches. The weights used by IMP-WFDR are the total relationship confidence, which is ad hoc. Future work will focus on identifying an optimal transformation of the network information into gene specific weights. We will also consider utilizing pathway information, which is contained alongside the IMP networks in the IMP webserver, to identify an optimal weighting scheme.

In conclusion IMP-WFDR is a powerful analytic tool that embraces state-of-the-art genetic network information in identifying differentially expressed genes in high-dimensional settings. We expect that IMP-WFDR and other tools that allow biologists to analyze high-throughut data in the context of biological relationships will play a key role in identifying genes differentially regulated in key models of human physiology.
